# The Constant or Galvanic Current of Electricity in Diseased of the Nervous System

**Published:** 1881-06-20

**Authors:** Edward C. Mann

**Affiliations:** New York City


### THE CONSTANT OR GALVANIC CURRENT OF ELECTRICITY IN DISEASES OF THE NERVOUS SYSTEM.

BY EDWARD C. MANN, M. D., NEW YORK CITY.

*Sciatica.*—In the treatment of sciatica, in common with other neuralgias, it is important to bear in mind that pathological causes which irritate the nerve high up in the trunk, produce pain at the peripheric distribution; and sensation excited by irritation of the origin or nucleus of a sensory nerve are uniformly referred to the periphery. The great predisposing cause of sciatica, in common with the other neuralgias, is heridetary predisposition, which results in the transmission of an imperfect central nervous system—a neurotic constitution. Sciatica is one of the most curable of neuralgias if properly treated. If injudiciously treated, it is often very intractable. We have as forms of sciatica, aside from a simple neuralgia, syphilitic and rheumatic forms of the disease, the former occurring very frequently, and in obstinate cases which have resisted all other treatment, we may get brilliant cures by giving iodide of potassium in rapidly increasing doses in combination with small doses of bichloride of mercury. The irritation set up by obstinate constipation, the puerperal state where the enlarged uterus produces an irritative pressure, or a tumor pressing on the nerve in the pelvis may all cause sciatica. The worst cases we meet with in practice occur between forty and fifty years of age. Primarily, rest is the great therapeutic agent. Our patient must not be allowed to walk, as muscular movements are very injurious, as the nerve is pulled upon by the muscles and the pain is thus aggravated.

Our patient must also be kept warm, and wear silk drawers if he can afford silk under-clothing, and the bowels kept carefully regulated. When the paroxysms of pain come on, we may alleviate them temporarily by hypodermic injections of morphia, and we may paralyze the sensibility of the peripheral nerves by local applications of aconite liniment, The use of the constant current of electricity by its stimulating and catalytic effects will enable us to get that perfect cure which should be our aim. The negative pole of a battery of 32 cells I place opposite the roots of the nerves which form the sciatic, and the positive pole is applied at the seat of pain. I make this application twice daily, and by keeping up the nutrition of the central nervous system at the same time, I obtain the most gratifying results even in cases of years' standing. The nutrition of the sciatic nerve is much improved and there is a heaithy change induced in the entire nerve. I have also cured some cases of sciatica by static electricity, using the Tœplar machine, charging the patient, and then drawing sparks along the course of the affected nerve by the wire brush.

In severe *lumbago*, affecting the dorsal muscles and the intercostals with severe, excruciating pains, making the patient bend almost double, I have experienced uniformly good results in every case from the use of the constant current to the affected region, together with slight ether inhalations. We certainly get a specific effect from the constant current in nearly all the neuralgias. In *facial neuralgia* I combine with the use ot electricity, gelseminum, or Dregnesnel's aconitia in 1-200 grain doses, gradually increased until physiological effects are produced. In *ovarian neuralgia* the relief to pain is sometimes magical from the constant current, and I also employ in the visceral neuralgias hypodermic injections of atropia in 1-100 to 1-50 grain doses. In all the neuralgias I am also in the habit of prescribing, with the most gratifying results, the solution of chloro-phosphide of arsenic in doses of 15 minims three times a day after meals. This most valuable remedy—the very best in cases of brain wasting from exhaustion—is prepared by bringing phosphorus and arsenic together in the presence of hydrochloric acid. It acts very beautifully in many diseases of the nervous system, improving brain power and sexual impetus, and produces no unpleasant symptoms.

In *paralysis*, from brain disease, particularly in hemiplegic cases where we find absence of any decided mental disturbance; slight thickness of speech; more or less deviation of the tip of the tongue to the paralyzed side when it is protruded; partial and incomplete paralysis of the facial muscles on the side on which the paralysis of the limbs exists; more or less complete loss of voluntary power over the left arm and leg (if the lesion is in or near the right corpus striatum); loss of sensibility and numbness on the paralyzed half of the body; and slight elevation of temperature on the paralyzed side—if the contractility of the muscles be perfect the use of electricity is contra-indicated.

When, in paralysis, we meet with the reactions of degeneration, wasting of muscles, or loss of normal muscular irritability or contractility, the galvanic or constant current is then indicated, and after we get, by this current, an irritability which responds to the induced or Faradic current, we may proceed with that current to the ultimate restoration or cure of paralysis. In hysterical paralysis, where the patient has no will to move her muscles, we may get a rapid and brilliant cure by the induced current.

In neuralgia or hyperæsthesia of the testes, which is a very painful neurosis, we have a perfect means of relief in the constant current of electricity conjoined with laxatives followed by tonics. In hyperæsthesia, or irritable state of the uterus, a very troublesome neurosis, we apply a cup-shaped electrode, attached to the negative pole, to the os uteri and the positive to the hypogastric or sacral region with uniform good results. Also local sedatives ʒi of morphia to ℥i of ungt. belladonna, and a little pill of this rolled up and introduced into the os. This is also a very valuable remedy in hysteria. The patient can hardly sit down, and coition is impossible in true hyperæthesia of the uterus. It results, I think, from neurasthenia. Nervous cardiac pain near the apex of the heart is a common and distressing neurosis, and this cardiac irritability is alleviated by centric galvanization. This form of application also relieves neuralgia or hyperæsthesia of the stomach, in which the vaso-motor nerves and the tone of the arteries are impaired. Spinal hyperæsthesia is also very amenble to treatment by the constant current. It should be remarked that neurotic pains of the spine are, as a rule, much more severe than those accompanying serious organic trouble. Neuralgia or hyperæsthesia of the breast in women is readily cured by the judicious use of the constant current. In cerebrasthenia or nervous prostration we have a hyperæsthesia or neuralgia of the entire brain; this condition may lead to insanity if not checked. We must give the patient rest and make him sleep, and feed him with milk, and not allow business cares or anxieties to worry him for at least one month.

The brain is enfeebled and hyperæsthesic, and the daily use of cephalic electrization will soon improve the nutrition and tone of the brain, and we shall cure our patient. In all cases build up and improve the nutrition of the central sensory nerve cells, as it is this condition of imperfect nutrition which causes neuralgia and hyperæsthesia. Cod-liver oil in small doses with the chloro-phosphide of arsenic in 15 minim doses after meals, and the daily use of the constant current in skillful hands will work wonders in many very severe cases. We have nerve exhaustion, a lowering of vital power in all these cases. We should always look for the cause of all these troubles, and we may perhaps light on some focus of irritation or a blood-poisoning.

In the early stage of progressive or general paralysis we may sometime gain great benefit from centric galvanization, and cut off the wearing impressions which are being transmitted practically without cessation to the brain.

In the incipient stages of insanity the constant current of electricity is of immense value, as it, if properly applied, antagonizes the various congestive states of the brain, which, unchecked, result in organic disease. I have spoken of this at length in my forthcoming work on "Psychological Medicine and allied Nervous Diseases."—*Lancet and Clinic.*

